# Effects of media campaign videos on stigma and attitudes towards treatment seeking for alcohol use disorder: a randomized controlled study

**DOI:** 10.1186/s12889-023-16811-4

**Published:** 2023-10-04

**Authors:** Sara Wallhed Finn, Anna Mejldal, Ruben Baskaran, Anette Søgaard Nielsen

**Affiliations:** 1 Unit of Clinical Alcohol Research, Institute of Clinical Research, University of Southern Denmark, J.B. Winsløws Vej 20, entrance. 220 B, Odense, 5000 Denmark; 2 Department of Global Public Health, Karolinska Institutet, Stockholm, Sweden; 3 SDU Health informatics and technology, Faculty of engineering, The Maersk Mc-Kinney Moller institute, University of Southern Denmark, Odense, Denmark; 4 Psychiatric Hospital, University Function, Region of Southern, Odense, Denmark

**Keywords:** Alcohol use disorder, Treatment seeking, Stigma, Video, Denmark

## Abstract

**Background:**

Alcohol Use Disorder (AUD) is one of the most stigmatized diagnosis, and stigma imposes a major barrier to treatment seeking. There is a need to develop interventions that can reduce stigma and increase treatment seeking. Little is known about the effects of video materials. The aim of this study was to investigate effects of different videos. The primary outcome was public stigma, and secondary outcomes were: self-stigma, and motivation to change own alcohol use; talking to someone else about their alcohol use; seeking information about AUD treatment or seeking AUD treatment.

**Methods:**

This is a three-armed double blind randomized controlled study. The study included 655 Danish adults. Data was collected at a study webpage, and the survey could be completed anywhere with Internet access. After informed consent and completing baseline measures, participants were randomized, 1:1:1 ratio, to a video (video 1 n = 228; video 2 n = 198; video 3 n = 229). Video 1 and 2 have been used in a national mass media campaign and video 3 was recorded for use in the present study. Immediately after exposure, follow-up measures were completed. Outcomes were analyzed with mixed effects linear regression.

**Results:**

In total n = 616 completed follow-up (video 1 n = 215; video 2 n = 192; video 3 n = 209). Randomization to video 1 and 3 decreased public stigma measured with “Difference, Disdain & Blame Scales”, while video 2 increased stigma. Video 2 compared to 1: 2.262 (95% CI 1.155; 3.369) *p* < 0.001. Video 3 compared to 1: -0.082 (95% CI -1.170; 1.006) *p* = 0.882. Video 3 compared to 2: -2.344 (95% CI -3.455; -1.233) *p* = 0.882. All videos reduced motivation to change own alcohol use. Participants with hazardous alcohol use, were more sensitive to the different videos, compared to low-risk alcohol use. Video 2 decreased motivation to seek information about treatment. No effects were seen on motivation to seek treatment, motivation to talk to someone else or self-stigma.

**Conclusions:**

Videos can have an immediate effect on level of public stigma. Other types of interventions are needed to increase motivation and reduce self-stigma. To avoid adverse effects in future interventions, the use of theoretical frameworks and stakeholder involvement is emphasized.

**Supplementary Information:**

The online version contains supplementary material available at 10.1186/s12889-023-16811-4.

## Background

Alcohol use disorder (AUD) is the most prevalent substance use disorder (SUD), directly affecting 100.4 million individuals worldwide [1]. In the EU, the one-year prevalence of alcohol dependence is estimated at 3.4% [2]. Globally, the total alcohol consumption is predicted to increase over the coming 10 years, which can lead to an increase in affected individuals and alcohol related consequences [3].

Even though AUD is common, it is among the most highly stigmatized psychiatric disorder in the Western world [4]. Stigma is a process where the majority population label a specific group in society, and where the labelling is associated with a perception of being different [5] and with negative stereotypes [6, 7]. Public stigma and self-stigma are two different aspects of the stigma process [8]. Public stigma consists of shared negative emotional responses and perceptions towards the labelled group, leading to discrimination. Self-stigma describes the process where an individual identifies themselves as belonging to the stigmatized group internalize the public stigma [9].

Stigma has a range of negative consequences for individuals with AUD. On a structural level, it can lead to discriminating policies and low financial allocations [10]. On an individual level, stigma hinders problem recognition for AUD and hampers recovery [11, 12]. Stigma also constitutes an important barrier to treatment-seeking [13–16]. It is well established that only a minority of individuals with AUD seek treatment [17, 18]. Denmark is no exception to this, even though treatment is readily accessible and free of charge [19]. Reducing stigma associated with AUD is therefore an important step to improve treatment seeking.

### The role of mass media campaigns

In Denmark, 98% of the population use the Internet, and 84% are active on social media [20]. It is therefore possible to reach a large proportion of the population via these channels. Mass media campaigns have the advantage of being easily implemented, with a high outreach. Mass media campaigns and unplanned mass media coverage, such as articles in newspapers about AUD, show a positive association with treatment-seeking in general and the use of self-help for reducing alcohol use [21, 22]. However, little attention has been given to its associations with treatment-seeking for AUD.

Interventions based on education and on increasing social contact between groups have been shown to be effective in reducing public stigma [23–25]. Mass media campaigns often include a personal story from someone with lived experience. This is an indirect form of contact, which can reduce stigma [26]. These stories can also reduce stigma by offering a broader perspective of the labelled outgroup and thereby increase tolerance and empathy [27, 28]. From current evidence it is, however, unclear whether mass media interventions can reduce public stigma. A Cochrane review concluded that mass media interventions may reduce prejudice, but there is insufficient evidence on the effects on discrimination [29]. However, none of the included studies in the review covered stigma associated with AUD.

Mass media campaigns have the potential both to reduce stigma and also increase treatment seeking for AUD. However, there is a dearth of knowledge on this topic.

### The RESPEKT campaign

The Danish NGO-organization “Alkohol & Samfund” [English: “Alcohol & Community”] and the private Foundation “Trygfonden” [aimed at supporting research and prevention-initiatives], in cooperation with media / PR agencies and local alcohol treatment services, have developed a media campaign, “RESPEKT,” which has been broadcast annually across Denmark since 2015. The aims of the campaign were to:

- Increase public awareness that the council offers AUD treatment free of charge.
- Increase the number of individuals who seek advice, or seek / initiate treatment for AUD.

The campaign is a targeted communication approach [30], where men in the general population age 40 to 70 years old with AUD are a primary target group, since they are considered the largest group of non-treatment-seeking individuals with AUD. Secondary target groups are the general population and the social network around individuals with AUD.

The campaign is multi-component, where the main channels are TV and Internet advertisements. The content of the main campaign video has varied over the years. From 2016 to 2018 and year 2020 the video showed three boys in a football locker room, where one boy proudly tells his friends that his father has stopped drinking. The video ends with father and son hugging, and the message “it gives respect to do something about your alcohol problem.” In 2019, the video showed a child at home, cleaning up beer cans while the father is asleep on the living room sofa. The boy makes sandwiches for the father and writes the number of the national telephone help line for AUD on a post-it note to his father, before leaving home.

The RESPEKT campaign is unique from an international perspective, with its aim to increase treatment-seeking. In a previously published cross-sectional study, we have studied how the message of the campaign was understood and how it impacted awareness, attitudes, and information seeking [31]. The results showed that the campaign was successful in evoking positive attitudes and strengthening the support for free treatment for AUD. However, due to the cross-sectional design in that study, it was not possible to disentangle effects of specific contents of the campaign. There is a need to deepen the knowledge about effects of different types of media content on stigma and treatment seeking, which recently also has been emphasized [32].

The aim of this study is to investigate effects of viewing either one of the two videos used in the RESPEKT campaign or a neutral video, informing that AUD treatment is free, on the primary outcome measure:

- public stigma.

The secondary outcome measures:

- motivation to change one’s own alcohol use.
- motivation to talk to someone else about their alcohol use.
- motivation to seek information about AUD treatment.
- motivation to seek AUD treatment.
- self-stigma.

The hypothesis is that randomization to video 1 with the boy and father at the football ground leads to lower level of public stigma and self-stigma, and also higher motivation to change one’s own alcohol use, to talk to someone else about their alcohol use, to seek information about treatment and seek treatment; compared to randomization to video 2 with the boy and father at home or viewing video 3, with information about AUD treatment. The rationale is that video 1 includes messages that can have a stigma reducing effect, as recovery, and messages that can increase motivation, via the focus on positive consequences of making a behavior change, as being a better parent [25, 33].

### Methods

#### Study-design

This is a three-armed double blind randomized controlled study.

#### Participants

The participants, adults 18 years and older, were recruited via convenience sampling.

#### Procedure

A study webpage was launched in year 2020, after the RESPEKT mass media campaign period was completed [31]. Information about the study webpage was given to individuals seeking information on the webpage Hope.dk, where information about alcohol, AUD and treatment are provided; on social media; to staff and students at the University of Southern Denmark; and to staff and patients in the addiction treatment services in Denmark. Study information was given on the webpage, informing that the aim of the project, was to learn about how people in general view treatment seeking for alcohol problems, in addition to stressing that participating in the study was voluntary. Also that those completing the task and questionaires within study could enter a lottery, where five gift baskets with a value of 400 DKK each were given away. After a Completely Automated Public Turing test to tell Computers and Humans Apart (CAPTCHA) to ensure humans and not bots participated, informed consent was obtained and the participants completed baseline measures. The participants were then randomized on a 1:1:1 ratio using the randomization function in REDCap [34] to one of three exposures:

1. The RESPEKT video featuring the boy and father at the football ground [35].
2. The RESPEKT video featuring the boy and father at home [36].
3. A video beginning: “Are you worried that you drink too much? Have others told you that, or are you concerned about your health?”, followed by information that AUD treatment is free of charge and that it is possible to seek anonymously [37]. This video was recorded as part of the current study, aiming to give neutral information similar as treatment services do to the general public. This video has not been used in any mass media campaigns.

Immediately after exposure the participants were asked to complete outcome measures.

### Data collection

Data was collected at the study webpage, and submitted to the REDCap database hosted by OPEN [34, 38], between 10th of March and 18th of November 2021. The participants could thus complete the survey in any physical place, with Internet access.

The baseline measures included:

- Demographic data on gender, age, education, marital status, children, country of birth and occupational status.
- Alcohol use, assessed with Alcohol Use Disorder Identification Test – consumption (AUDIT C), including three items on level of alcohol use which were scored 0–4, giving a total score ranging from 0 to 12 [1]. Total scores of three or above for women and four and above for men indicate hazardous alcohol use [39].
- Previous experience of seeking treatment for AUD.
- Knowing someone with experience of AUD.
- Public stigma assessed with Difference, Disdain & Blame Scales for Public Stigma [40, 41]. The questionnaire includes nine items rated on a scale from one (Not at all) to nine (Very much), giving a total score from 9 to 81.
- Motivation to change one’s own alcohol use, motivation to talk to someone else about their alcohol use, motivation to seek information about AUD treatment and motivation to seek AUD treatment were assessed with a visual analogue scale (VAS), ranging from one (Not at all) to nine (To a very high degree) [42].

Participants who endorsed ongoing or previous concern for their alcohol use were presented a questionnaire measuring self-stigma, the Self-Devaluation Subscale, of the Substance Abuse Stigma Scale [43]. The questionnaire includes eight items rated from one (Never) to five (Very often), giving a total score ranging from 8 to 40.

Immediately after exposure, awareness of seeing the video previously was assessed and then measures of public stigma, self-stigma and motivation were repeated.

Which video, participants were randomized to, was blinded to the researchers and only a number (video 1, 2 or 3) was presented.

There was no a priory power calculation.

### Data analyses

Characteristics of participants at baseline were reported for the overall sample and separately for the three exposure groups. Counts and proportions were reported for categorical characteristics.

The numerical data on public stigma, motivation to change one’s own alcohol use, motivation to talk to someone else about their alcohol use, motivation to seek information about AUD treatment, motivation to seek AUD treatment and self-stigma were analyzed by mixed effects linear regression. The models included a fixed effect for group randomized to, a fixed effect for time point (baseline and follow-up) and a fixed effects interaction between group and time point. As baseline measurements were obtained before randomization, groups at baseline were modelled as a separate common treatment category, constraining baseline measurements to no systematic treatment effect between the groups. All mixed models included a random intercept for each participant. Normality assumptions were evaluated visually, and deviations were handled by repeating the analysis with nonparametric bootstrapping. Using the maximum likelihood estimator and assuming the dropout mechanism is ‘missing at random’, linear mixed models deal efficiently with missing values. Estimates were calculated with 95% confidence intervals (CI). Cohen’s *d* was calculated within groups from means and standard deviations at the pre and post measure for statistically significant results.

As the results on motivation to change own alcohol use showed the opposite result from a priori hypothesis, post hoc analyses were performed to understand the findings more fully. First, the sample was stratified into two, according to level of motivation at baseline, where the participants who scored in 75th percentile or higher were grouped into the high motivation-group and compared to the participants who scored in the 74th percentile and lower. Second, the sample was stratified according to level of alcohol use according to the AUDIT-C – one group with participants with low-risk alcohol use, and one group with participants with hazardous alcohol use.

All analyses were carried out using Stata MP 16.1 (StataCorp LP, College Station, TX). The blinding of which video the participants saw, was not broken until the analyses were completed.

## Results

**Fig. 1:**
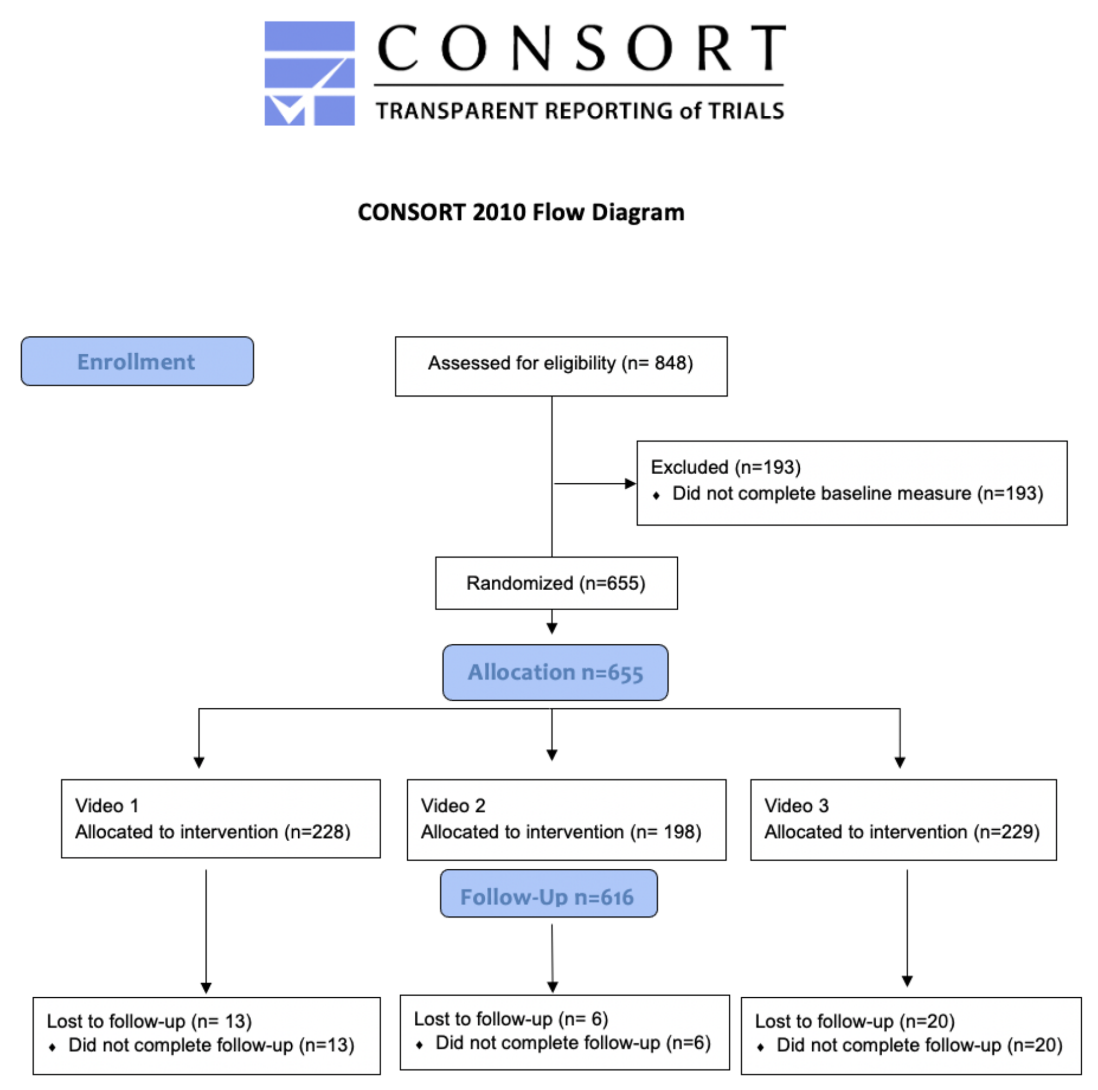
Consort flow diagram

Of 848 participants who entered the study, 72% (n = 655), completed the baseline measures and were randomized (Fig. 1). Among those randomized, 94% (n = 616) completed follow up measures. Of those randomized to video 1 (the football ground), 5.8% were lost to follow up, among participants randomized to video 2 (at home), the proportion was 3.1% and among those randomized to video 3 (information) it was 8.8%. Thus, fewer participants randomized to video 2 were lost to follow up, chi2 [2] = 6.13 *p* = 0.047.

The majority of participants were female (75%) and the mean age was 43 (SD 14). A majority, 68%, had an employment, while circa 20% were students (Table 1). 80% had more than 12 years of education. The vast majority were born in Denmark. Circa half had hazardous alcohol use according to AUDIT-C, 7% had previously sought treatment for AUD and nearly 90% knew someone with AUD. Almost every fourth participant (22%) had ongoing or previous concerns about their own level of alcohol use.

**Table 1:** Demographic of participants who completed the follow up measures

			Video		
		1	2	3	Total
**Total**	n	228	198	655	655
	%	34.8	30.2	35.0	100.00
**Sex**					
Female	n	179	144	171	494
	%	78.5	72.7	74.7	75.4
Male	n	49	53	58	160
	%	21.5	26.8	25.3	24.4
Other	n	0	1	0	1
	%	0.0	0.5	0.0	0.2
**Age category**					
18–30 years	n	69	49	50	168
	%	30.3	24.8	21.9	25.7
31–49 years	n	81	78	88	247
	%	40.9	39.4	38.4	37.7
50–79 years	n	78	71	91	240
	%	34.2	35.9	39.7	36.6
**Occupation**					
Working	n	147	138	162	447
	%	64.5	69.7	70.7	68.2
Student	n	55	45	38	138
	%	24.1	22.7	16.6	21.1
Pensioner, at-home, free or other	n	25	15	28	68
	%	11.0	7.6	12.2	10.4
Missing	n	1	0	1	2
	%	0.4	0.0	0.4	0.3
**Martial status**					
Married, co-habiting or in a relationship	n	164	150	172	486
	%	71.9	75.8	75.1	74.2
Single	n	64	48	57	169
	%	28.1	24.2	24.9	25.8
**Having children**					
Yes	n	149	128	160	437
	%	65.4	64.6	69.9	66.7
No	n	79	70	68	217
	%	34.6	35.4	29.7	33.1
Missing	n	0	0	1	1
	%	0.0	0.0	0.4	0.2
**Education**					
up to 12 years	n	41	44	43	128
	%	18.0	22.2	18.8	19.5
> 12 years	n	186	154	186	526
	%	81.6	77.8	81.2	80.3
Missing	n	1	0	0	1
	%	0.4	0.0	0.0	0.2
**Country of birth**					
Denmark	n	213	185	216	614
	%	93.4	93.4	94.3	93.7
Europe (excluding Denmark)	n	12	9	7	28
	%	5.3	4.6	3.1	4.3
Other country	n	3	4	6	13
	%	1.3	2.0	2.6	2.0
**Hazardous alcohol use (yes)** ^**1)**^	n	109	99	114	322
	%	47.8	50.5	50.4	49.5
**Previous treatment for AUD**					
No	n	211	187	210	608
	%	92.5	94.4	91.7	92.8
Yes	n	17	11	18	46
	%	7.5	5.6	7.9	7.0
Missing	n	0	0	1	1
	%	0.0	0.0	0.4	0.2
**Know someone with AUD**					
No	n	34	20	24	78
	%	14.9	10.1	10.5	11.9
Yes, one person	n	54	35	43	132
	%	23.7	17.7	18.8	20.2
Yes, several persons	n	140	143	162	445
	%	61.4	72.2	70.7	67.9
**Do you think you drink too much [alcohol]?**					
No	n	171	151	188	510
	%	75.0	76.3	82.1	77.9
No, but have previously	n	33	28	27	88
	%	14.5	14.1	11.8	13.4
Yes	n	24	19	14	57
	%	10.5	9.6	6.1	8.7

1) Alcohol use, assessed with AUDIT C, total score ranging from 0 to 12. Total scores of three or above for women and four and above for men indicate hazardous alcohol use

The time for completing the survey did not differ between groups chi2 [2] = 0.300 *p* = 0.861 (Table 2).

**Table 2:** Survey duration and proportion of the participants who endorsed seeing the campaign film before the study

				Film			
			1	2	3	Total	
**Survey duration** (minutes.seconds)		median	6.4	6.5	6.3	6.4	
**Video duration** (minutes.seconds)			0.30	0.53	0.46	n/a	
**Can you remember seeing this film before?**							
Yes		n	160	36	5	201	
		%	74.4	18.8	2.4	32.6	
No		n	50	152	199	401	
		%	23.3	79.2	95.2	65.1	
Don’t know		n	5	4	5	14	
		%	2.3	2.1	2.4	2.3	

Awareness varied between which video the participants were randomized to. Almost three out of four endorsed having seen video 1 before this study, one in five endorsed having seen video 2, while only 2% endorsed having seen video 3 before, chi2 [4] = 277.79 *p* < 0.001.

The mean score on public stigma was 33.28 (SD 10.22, median 32) at baseline (Table 3). The vast majority endorsed a low motivation to change their alcohol use (mean 2.34, SD 2.18, median 1), to seek information about treatment for AUD (mean 3.35, SD 2.68, median 2) and to seek treatment for AUD (mean 1.47, SD 1.49, median 1). The participants endorsed comparatively higher motivation to talk to someone else about their alcohol use (mean 4.76, SD 2.74, median 5). At baseline, the mean score of self-stigma was 14.12 (SD 7.06, median 11.5).

**Table 3:** Mean, SD and median of each variable, at pre and post measure, presented according to randomization group

			Video 1			Video 2			Video 3	
		Mean	SD	Median	Mean	SD	Median	Mean	SD	Median
Public stigma	pre	33.71	10.11	33	31.94	9.56	31	34.04	10.80	31
Public stigma	post	32.52	10.24	32	33.25	10.98	32	32.66	10.82	31
Motivation to change own alcohol use	pre	2.30	2.20	1	2.52	2.26	1	2.22	2.08	1
Motivation to change own alcohol use	post	2.04	2.12	1	1.81	1.83	1	1.89	1.98	1
Motivation to talk to someone else about their alcohol use	pre	4.58	2.77	5	4.83	2.75	5	4.89	2.71	5
Motivation to talk to someone else about their alcohol use	post	4.49	2.85	5	4.63	2.87	4	4.76	2.89	5
Motivation to seek information about treatment for AUD	pre	3.10	2.58	2	3.41	2.58	2	3.54	2.83	2
Motivation to seek information about treatment for AUD	post	3.05	2.66	2	3.09	2.67	2	3.38	2.92	2
Motivation to seek treatment for AUD	pre	1.48	1.48	1	1.46	1.42	1	1.48	1.57	1
Motivation to seek treatment for AUD	post	1.42	1.49	1	1.40	1.37	1	1.50	1.64	1
Self-stigma (n = 138)	pre	14.13	6.58	12	14.04	6.90	13	14.21	8.05	10
Self-stigma (n = 66)	post	15.93	8.19	12	16.44	7.76	14.50	19.28	9.35	18.50

**Fig. 2:**
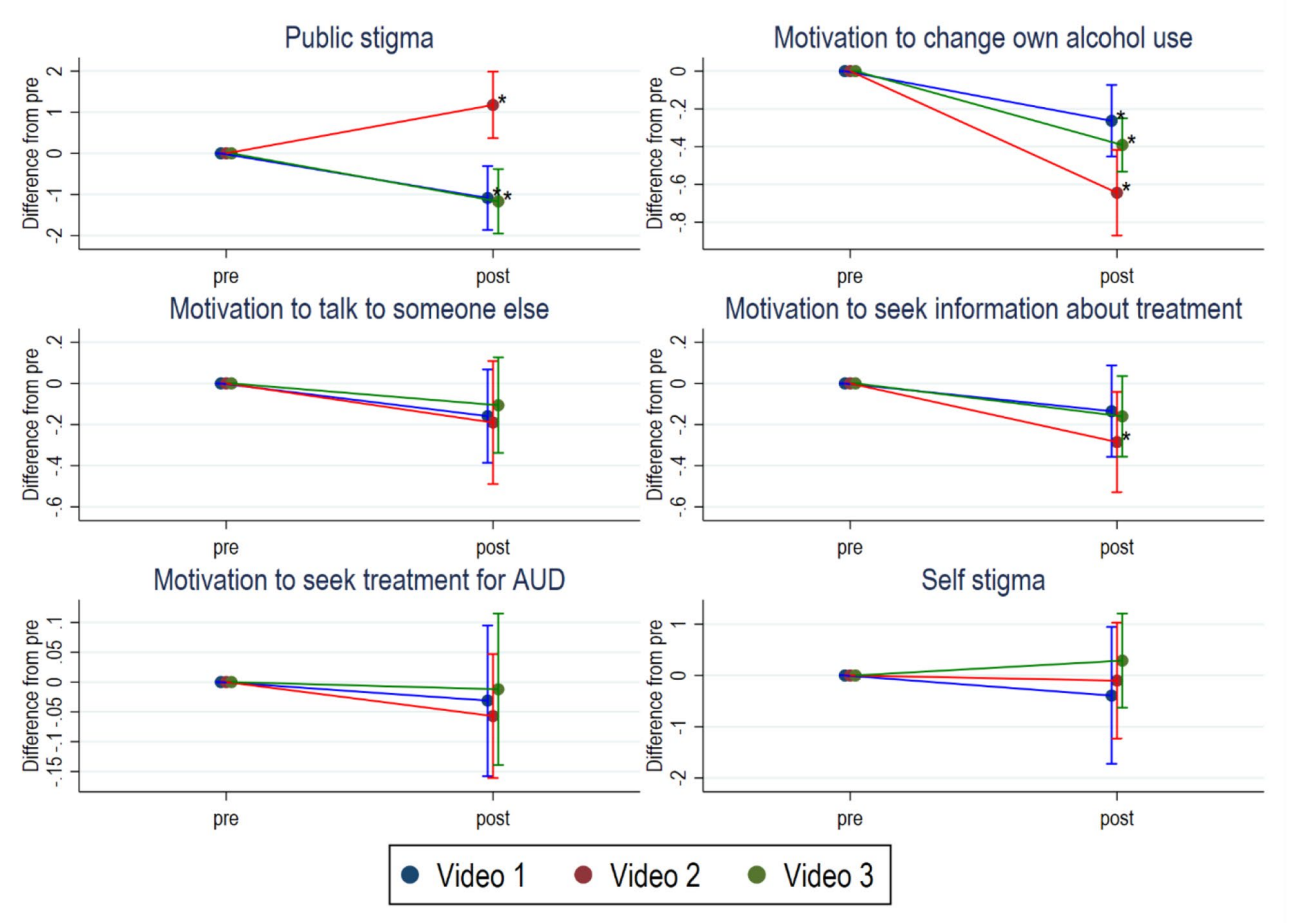
Estimates of change from pre to post measure presented according to randomization with 95% CI

Among participants randomized to video 1 and 3, a decrease in public stigma was seen from baseline to follow-up (Fig. 2). For video 1 Cohen’s *d* within group was − 0.117 and for video 3 -0.035. The opposite – an increase in public stigma – was seen among participants randomized to video 2, Cohen’s *d* 0.127. At follow up, participants randomized to video 2 had higher level of public stigma compared to those randomized to video 1 or 3. There was no difference between video 1 and 3.

In all three conditions, a decrease in motivation to change own alcohol use was seen from pre to post measure. There was a larger decrease in motivation among participants randomized to video 2 compared to video 1. Cohen’s *d* within group for video 1 was − 0.120; for video 2 -0.345; and for video 3 -0.163.

Among participants randomized to video 2, a decrease in motivation to seek information about treatment for AUD was seen from baseline to follow-up, with Cohen’s *d* -0.122. No difference between groups was found.

No change in motivation to talk to someone else, motivation to seek treatment for AUD or self-stigma was found.

### Post hoc analyses

#### Motivation to change own alcohol use – grouped according to level of motivation

All groups, but participants with low motivation at baseline randomized to group 1, decreased their motivation to change one’s alcohol use from pre to post measure (Table 4).

**Table 4:** Post hoc analyses of within and between group differences in motivation to change one’s own alcohol use. Participants grouped according to level of motivation (low or high)

	Group 1 Within group		Group 2 Within group		Group 3 Within group	
	Coeff (95% CI)	*p*	Coeff (95% CI)	*p*	Coeff (95% CI)	*p*
Low motivation (n = 467)	-0.014 (-0.083; 0.056)	0.698	-0.120 (-0.185; -0.055	0.000*	-0.082 (-0.130; -0.034)	0.001*
High motivation (n = 176)	-0.958 (-1.537; -0.378)	0.001*	-1.883 (-2.466; -1.301)	< 0.001*	-1.241 (-1.704; -0.777)	< 0.001*
	Group 2 − 1 Between groups		Group 3 − 1 Between groups		Group 3 − 2 Between group	
	Coeff	*p*	Coeff	*p*	Coeff	*p*
Low motivation (n = 467)	-0.106 (-0.194; -0.019)	0.017*	-0.068 (-0.150;0.014)	0.102	0.038 (-0.039; 0.116)	0.330
High motivation (n = 176)	-0.926 (-1.727; -0.124)	0.024*	-0.283 (-1.018; 0.452)	0.450	0.643 (-0.124; 1.409)	0.100

Randomization to video 2 decreased motivation to change alcohol use to a larger extent compared to randomization to video 1. The same findings were made both among participants with low and high motivation at baseline. No other group differences were found.

### 1.3.2. Post hoc analyses

#### Motivation to change own alcohol use – grouped according to level of alcohol use

There was a decrease in motivation to change one’s alcohol use in all groups, except among participants with low-risk alcohol use randomized to video 1 (Table 5).

**Table 5:** Post hoc analyses of within and between group differences in motivation to change one’s own alcohol use. Participants grouped according to level of alcohol use (low-risk or hazardous alcohol use)

	Group 1 Within group		Group 2 Within group		Group 3 Within group	
	Coeff (95% CI)	*p*	Coeff (95% CI)	*p*	Coeff (95% CI)	*p*
Low risk alcohol use (n = 333)	-0.210 (-0.477; 0.057	0.124	-0.288 (-0.561; -0.015	0.039*	-0.116 (-0.205; -0.028)	0.010*
Hazardous alcohol use (n = 322)	-0.303 (-0.544; -0.061)	0.014*	-0.983 (-1.327; -0.639)	< 0.001*	-0.675 (-0.928; -0.422)	< 0.001*
	Group 2 − 1 Between groups		Group 3 − 1 Between groups		Group 3 − 2 Between group	
	Coeff	*p*	Coeff	*p*	Coeff	*p*
Low risk alcohol use (n = 333)	-0.078 (-0.452; 0.295)	0.681	0.093 (-0.180;0.366)	0.503	0.172 (-0.108; 0.451)	0.229
Hazardous alcohol use (n = 322)	-0.680 (-1.095; -0.265)	0.001*	-0.373 (-0.724; -0.022)	0.037*	0.307 (-0.117; 0.731)	0.155

Among participants with hazardous alcohol use, those randomized to group 2 reported lower motivation to change compared to those randomized to group 1. Participants with hazardous alcohol use randomized to group 3 reported lower motivation compared to those randomized to group (1) No differences were found between participants with hazardous alcohol use randomized to group 3 and group (2) No differences between groups were found among participants with low-risk alcohol use.

## Discussion

### Level of public stigma

The a priori hypothesis, that ***level of public stigma*** would be lower after randomization to video 1, was partially confirmed. Video 1, depicting the boy and the father at the football ground, and video 3, with information, decreased public stigma. This is in line with previous studies showing that exposure to mass media campaign materials can reduce public stigma [23]. The opposite - an increase - in public stigma was seen among participants randomized to video 2, with the boy and father at home. This study found changes within groups corresponding to very small to small effect sizes, which is similar compared to other interventions aiming to reduce public stigma [48]. However, even small changes that are scaled up can contribute to meaningful changes on a population level.

Video 1 is set in a context common for many in the general Danish population - children and parents at football. This can potentially question the stereotype that individuals with AUD are different from others [5]. The hypothesized mechanism of action is that reducing the perception of differentness can increase empathy and lower anger towards the labelled outgroup, leading to a decrease in disdain [41]. Another message in video 1 is that it “gives respect” to make a change, and the father is in recovery from AUD. Recovery is a message that has shown to decrease public stigma [33]. However, the message that recovery means abstinence can reinforce the perception that treatment services only allow abstinence as a treatment-goal. This perception is an important barrier to treatment seeking [44], even though it is well known that recovery from AUD is more diverse [45]. Future studies should investigate how different narratives of recovery affect stigma.

Video 2 depicted a father with probably severe AUD, asleep on a sofa. While the message of the video is that significant others to problem drinkers should be helped, the video also includes several messages that can strengthen the stereotype of differentness – not being in recovery [33] and being unreliable as a parent [46], which can contribute to the increase in stigma. This finding emphasizes the importance of careful piloting of video materials, using a theoretical framework in the developmental phase, to avoid causing undesired effects.

Video 3 included information about AUD treatment and included concerns about alcohol use commonly reported in the general population [47]. The video did not make an emotional appeal but rather gave factual information in a neutral tone and language. This could reduce the perception of differentness [5]. Moreover, the message that treatment is effective can signal the possibility of recovery, and thus explain the decrease in stigma [33].

### Motivation to change one’s own alcohol use

Overall, the ***motivation to change one’s alcohol use*** was low among the participants at baseline and even lower after randomization to the videos. Post hoc analyses showed participants with hazardous alcohol use, compared to those with low-risk alcohol use, seemed more sensitive to the different videos, where randomization to videos 2 and 3 had a more detrimental effect on motivation compared to video 1.

Available evidence on both mass media campaigns aiming to reduce alcohol and tobacco harm, show that campaign messages including reasons to make a change, so called “why-messages”, and messages that address long-term harms of alcohol, are more effective in strengthening motivation to change, compared to campaign messages focusing on *how* to make the change [48–50].

Video 1, which did not decrease motivation to the same extent as the other two videos, included aspects of “why-messages”, as being a present parent and gaining respect from others. However, these social consequences do not seem as effective in eliciting motivation as messages on long-term and internal physical harm of alcohol use, which apply broader to all viewers [48]. All three videos in the present study were set in a real-world context, whereas evidence from the alcohol harm literature suggests that animated messages elicit the highest motivation to change [48]. Especially regarding alcohol, it might be important to use messages applicable to all viewers, rather than targeted communication [30]. As AUD is highly stigmatized, targeted communication can possibly trigger “label avoidance” [51]. That means that hazardous alcohol users can distance themselves from identifying as problem drinkers in order to avoid being stigmatized, which prevent them from identifying with the campaign message [12].

Future studies should also consider the emotional tone of videos. The alcohol harm literature suggests including a negative emotional tone, compared to a positive, elicit higher motivation to change [48, 52]. Our study suggests the opposite. There may be important differences in mechanism targeting reducing alcohol related harm compared to increasing treatment seeking.

### Motivation to seek treatment for AUD

Participants randomized to video 2, decreased their ***motivation to seek treatment for AUD***, which possibly could be attributed to a decrease in problem recognition after exposure to the video with the father with the severe AUD. No change was seen after randomization to the other two conditions. These results are in line with results from our previous study of the RESPEKT campaign, where only 2% of those exposed to the campaign videos self-reported seeking more information about AUD treatment [31].

A recent cross-sectional survey showed a positive association between engaging in social media posts of others change of alcohol use or treatment seeking for AUD, and own self-reported treatment seeking [32]. Specifically, the perception that AUD treatment is effective was associated with treatment seeking. Video 3 in the present study, included the message that AUD treatment is effective, but did not elicit motivation to seek treatment. It is possible the message would have needed to be communicated differently to have an effect. For example, as a first-person message, which has been suggested to normalize help seeking [53]. A rather large group of individuals engage in video materials, as TikTok, to support their SUD recovery [54]. However, knowledge about the effects of videos on eliciting behavior change is scarce. In the present study, video 2 had fewest participants lost to follow up, suggesting the video is engaging, but it clearly does not elicit the intended behavior change.

### Motivation to talk to someone else about their alcohol use

The social network around individuals with AUD was a secondary target group of the RESPEKT campaign. Social support is an important factor for seeking AUD treatment [55–57]. To increase support for those seeking help, at a population level, holds great potential for narrowing the treatment gap. However, none of the three videos affected ***motivation to talk to someone else about their alcohol use***, suggesting that other interventions are needed to achieve this. Community Reinforcement and Family Training (CRAFT) is an evidence-based program aiming for this goal [58], and have been found effective [59–61]. Programs like CRAFT are typically offered at a specialist care level, and there is a need for interventions aimed at a broader target group. Since the communication component in CRAFT has been reported to be particularly helpful [62], communication may be a suitable skill to strengthen on a population level.

### Self-stigma

Nor were any effects on **self-stigma** found. Thus far, interventions that have shown to reduce self-stigma have been psychological based on acceptance and commitment therapy, delivered individually or in group [24]. However, this type of intervention is expensive and difficult to scale up. One path for future research is to evaluate self-help interventions to reduce self-stigma [63].

### Strengths and limitations

The use of self-report measures, can pose an increased risk of socially desirable answers. A stigma questionnaire measuring differentness was used, which is considered to impose less risk of bias [64, 65]. The other outcomes were measured with items on motivation, which even though not directly measuring the intended behavior outcome, has shown to predict later behaviors [66]. In future studies, we suggest using objective data from national registers to measure treatment-seeking behaviors.

Moreover, the study focused on immediate effects of randomization to different videos. This exposure may not be transferable to a real-world context, where exposure to campaign videos can occur repeatedly. Future studies should include a longer follow up, especially for interventions aiming to reduce stigma [67].

The level of public stigma associated with AUD reported in this study, was lower compared to a recent study which included participants from the general Danish population [68], which possibly is associated either with the large proportion of participants who reported own hazardous alcohol use or endorsed knowing someone with AUD. This may be considered another limitation of the study and is probably due to the use of a convenience sample, including only a minority of participants who endorse concern for their own level of alcohol use. It could also be related to the use of AUDIT-C, which imposes an elevated risk of false positive cases among those screening positive [69]. This method of recruitment did only to a small extent reach the primary target group for the RESPEKT campaign, men 40 years or older with AUD. Also, very few participants were born outside of Denmark.

An important strength of the study is the double-blinded design, where the participants were blinded for the randomization and the researchers for the allocation, which ensures validity by minimizing observer bias and researcher bias. Also, that the study is conducted by independent researchers, not involved in the development of the campaign, which can contribute with new perspectives and insights.

## Conclusions

Video materials focusing on recovery and reducing the perception of differentness have an immediate effect on decreasing public stigma associated with AUD. The unexpected detrimental effects highlight the need for careful considerations, use of theoretical frameworks and stakeholder involvement in the development of video materials. There is a need to develop other types of messages or interventions to increase motivation to seek treatment for AUD, motivation to talk to someone else and reduce self-stigma. We suggest future studies focus on messages aimed to the general population rather than targeted communication.

### Electronic supplementary material

Below is the link to the electronic supplementary material.

Supplementary Material 1

## Data Availability

The data underlying this article cannot be shared publicly due to the privacy of individuals that participated in the study. The data will be shared on reasonable request to the corresponding author.

## List of abbreviations

AUD: Alcohol Use Disorder
AUDIT C: Alcohol Use Disorder Identification Test Consumption
CAPTCHA: Completely Automated Public Turing test to tell Computers and Humans Apart
CI: Confidence Intervals
CRAFT: Community Reinforcement and Family Training
SUD: Substance Use Disorders
VAS: Visual Analogue Scale
