# Supplementary material for: Effects of media campaign videos on stigma and attitudes towards treatment seeking for alcohol use disorder: a randomized controlled study
Source: BMC Public Health. 2023 Oct 4;23:1919. doi: 10.1186/s12889-023-16811-4 (PMC10552234; doi:10.1186/s12889-023-16811-4)
Supplement: Supplementary file 1 — Supplementary Material 1 [file 12889_2023_16811_MOESM1_ESM.docx]

Appendix

Estimates of change within groups from pre to post measure presented according to randomization (presented in Figure 2)

|  | Group 1  Within group | | Group 2  Within group | | Group 3  Within group | |
| --- | --- | --- | --- | --- | --- | --- |
|  | Coeff (95% CI) | *p* | Coeff (95% CI) | *p* | Coeff (95% CI) | *p* |
| Public stigma | -1.085  (-1.862; -0.309) | 0.006* | 1.177  (0.369; 1.985) | 0.004* | -1.167  (-1.951; -0.384) | 0.003* |
| Motivation to change own alcohol use | -0.263  (-0.452; -0.073) | 0.007* | -0.644  (-0.871; -0.417) | 0.000* | -0.391  (-0.532; -0.250) | <0.001* |
| Motivation to talk to someone else | -0.159  (-0.386; 0.068) | 0.169 | -0.190  (-0.489; 0.109) | 0.213 | -0.106  (-0.338; 0.127) | 0.373 |
| Motivation to seek information about treatment | -0.135  (-0.357; 0.088) | 0.236 | -0.285  (-0.529; -0.041) | 0.022* | -0.160  (-0.356; 0.036) | 0.110 |
| Motivation to seek treatment for AUD | -0.031  (-0.158; 0.095) | 0.629 | -0.057  (-0.161; 0.047) | 0.283 | -0.012  (-0.139; 0.115) | 0.854 |
| Self stigma | -0.392  (-1.723; 0.945) | 0.565 | -0.101  (-1.230; 1.028) | 0.861 | 0.289  (-0.629; 1.207) | 0.537 |

Appendix

Estimates of change between groups from pre to post measure presented according to randomization (presented in Figure 2)

|  | Group 2 - 1  Between groups | | | Group 3 - 1  Between groups | | Group 3 - 2  Between group | |
| --- | --- | --- | --- | --- | --- | --- | --- |
|  | Coeff  (95% CI) | | *p* | Coeff (95% CI) | *p* | Coeff (95% CI) | *p* |
| Public stigma | 2.262  (1.155; 3.369) | | <0.001* | -0.082  (-1.170; 1.006) | 0.882 | -2.344  (-3.455; -1.233) | <0.001* |
| Motivation to change own alcohol use | -0.382  (-0.669; -0.094) | | 0.009* | -0.129  (-0.360; 0.103) | 0.276 | 0.253  (-0.018; 0.524) | 0.068 |
| Motivation to talk to someone else | -0.031  (-0.405; 0.343) | | 0.872 | 0.054  (-0.271; 0.378) | 0.746 | 0.084  (-0.284; 0.453) | 0.653 |
| Motivation to seek information about treatment | -0.150  (-0.471; 0.170) | | 0.359 | -0.250  (-0.318; 0.268) | 0.867 | 0.125  (-0.189; 0.439) | 0.435 |
| Motivation to seek treatment for AUD | | -0.026  (-0.186; 0.135) | 0.755 | 0.019  (-0.155; 0.193) | 0.828 | 0.045  (-0.117; 0.206) | 0.585 |
| Self stigma | | 0.292  (-1.458; 2.041) | 0.744 | 0.681  (-0.959; 2.322) | 0.416 | 0.390  (-1.038; 1.818) | 0.593 |
